# Upper limb isokinetic muscle strength predicts the performance in cross-country sit-skiing

**DOI:** 10.1038/s41598-022-10103-4

**Published:** 2022-04-12

**Authors:** Chenglin Liu, Yuan Tian, Longfeng Zhou, Zhulin Tian, Gang Sun, Jun Yin, Zhixiong Zhou

**Affiliations:** 1 Sports Artificial Intelligence Institute, Capital University of Physical Education and Sports, No. 11 North Third Ring Road West, Beijing, 100191 People’s Republic of China; 2 School of Physical Education and Coaching Science, Capital University of Physical Education and Sports, No. 11 North Third Ring Road West, Beijing, 100191 People’s Republic of China

**Keywords:** Occupational health, Public health

## Abstract

The double poling (DP) technique in cross-country sit-skiing is primarily considered as an upper-body exercise. The upper limb muscle strength and motion economy are important factors accounting for DP performance in cross-country sit-skiing. The present study investigates how upper limb muscle strength predicts DP performance in cross-country sit-skiing. A total of 19 female non-disabled college students (age 23.2 ± 0.8 years, BMI 20.4 ± 2.2) performed 30-s and 3-min DP performance tests using a sit-skiing ergometer. Isokinetic muscle strength of the shoulder and elbow extensor were measured at the angular velocity of 30°/s, 60°/s, and 120°/s with an ISOMED2000 isokinetic system. A medium correlation was found between DP output power and isokinetic upper limb muscle strength (shoulder strength at all speeds, *r* = 0.39–0.74, *p* ≤ 0.1). Multiple regression analyses which were employed to predict power production in the 30-s and 3-min tests showed that shoulder extension strength at 60°/s accounted for 34% of the variation in the 30-s test, and 40% of the variance in the 3-min test. Muscle strength and biomechanical analysis of DP process indicated that upper limb extensor muscle strength and muscle coordination were important factors for the power output generation in sit-skiing DP. These results may use to guide special physical fitness training for paralympic cross-country sit-skiing.

## Introduction

Paralympic cross-country sit-skiing is an aerobic endurance sport, which requires athletes to sit on a sit-ski and generate propulsion with upper limbs. The pushing technique performed by sit-skiers is adopted from the double poling (DP) technique used by standing non-disabled skiers. The determinants of cross-country skiing usually include technical skill, muscle strength, and aerobic capacity. Previous studies used video analysis of kinematic parameters obtained both during the race^1^ and in the lab^2^ so as to determine the most economical technical action, biomechanical analysis of muscle usage in the DP technique^3^, and correlation analysis between DP performance and kinetic factors^4^, muscular strength variables^5^, and energetic cost^6^. Accumulated evidences have shown that the upper body power (UBP) has an important role in cross-country ski racing^7,8^, which was further confirmed by statistical analysis from both aerobic energy systems^9^ and shorter sprint-type UBP tests^10^. However, the required physical fitness for performing DP in cross-country sit-ski still remains unclear and needs to be urgently analyzed.

The isokinetic muscle strength test is the most commonly used method to assess muscle strength in limbs^11,12^. Also, the positive relationship between isokinetic muscle strength and athletic ability has been well established^13,14^. To further elucidate the relationship between physical fitness and cross-country sit-skiing performance, it is necessary to measure and analyze the isokinetic muscle strength of upper limb muscle groups.

Previous studies have discussed the relationship between muscle strength and DP performance, where muscle strength was represented by one-repetition maximum (1-RM) strength^15^ or lean mass^16^. Yet, isokinetic muscle strength is rarely mentioned in the DP technical analysis as the normative standard for evaluating muscle strength^17^. Limited biomechanical data are available on the relationship between upper limb isokinetic muscle strength and DP performance of cross-country sit-skiing.

The aim of the current study was to examine the relationship between the specific muscle strength of upper limb muscle groups and the power output in cross-country sit-skiing. Isokinetic muscle strength of shoulder and elbow were measured with a isokinetic system, and DP of sit-skiing was performed on a cross-country sit-skiing ergometer. The reported results may be used to guide special physical fitness training for paralympic cross-country sit-skiing.

## Results

A total of 19 female non-disabled college students majoring in physical training with a mean age of 23.2 ± 0.8 years, a height of 162.9 ± 4.3 cm, and a BMI of 20.4 ± 2.2, were included in the study, as shown in Table 1. The measured isokinetic muscle extension strength at low speed was larger than at high speed, both in shoulder and elbow joints. In the tests of 30-s and 3-min, the poling distance of the poling phase was similar (0.84 m and 0.81 m, respectively), and the variance of the two data was small (SD = 0.1 m).

**Table 1:** Demographics, strength, and output power measures (n = 19).

	Mean	SD
Age (years)	23.2	0.8
Height (cm)	162.9	4.3
Weight (kg)	54.2	5.3
Shoulder muscle strength (N*m)		
Extend, 30°/s	31.0	8.3
Extend, 60°/s	30.1	7.9
Extend, 120°/s	22.8	7.5
Elbow muscle strength (N*m)		
Extend, 30°/s	21.8	5.9
Extend, 60°/s	19.5	4.9
Extend, 120°/s	16.0	3.6
**Distance of a poling phase (m)**		
30-s ∆s	0.84	0.1
3-min ∆s	0.81	0.1
**Output power (J)**		
30-s power	202.3	39.2
3-min power	1087.0	295.2

*SD* standard deviation, ***∆****s* distance of a poling phase during double poling.

### Correlations among technical performance, output power, and upper limb isokinetic muscle strength

**Figure 1:**
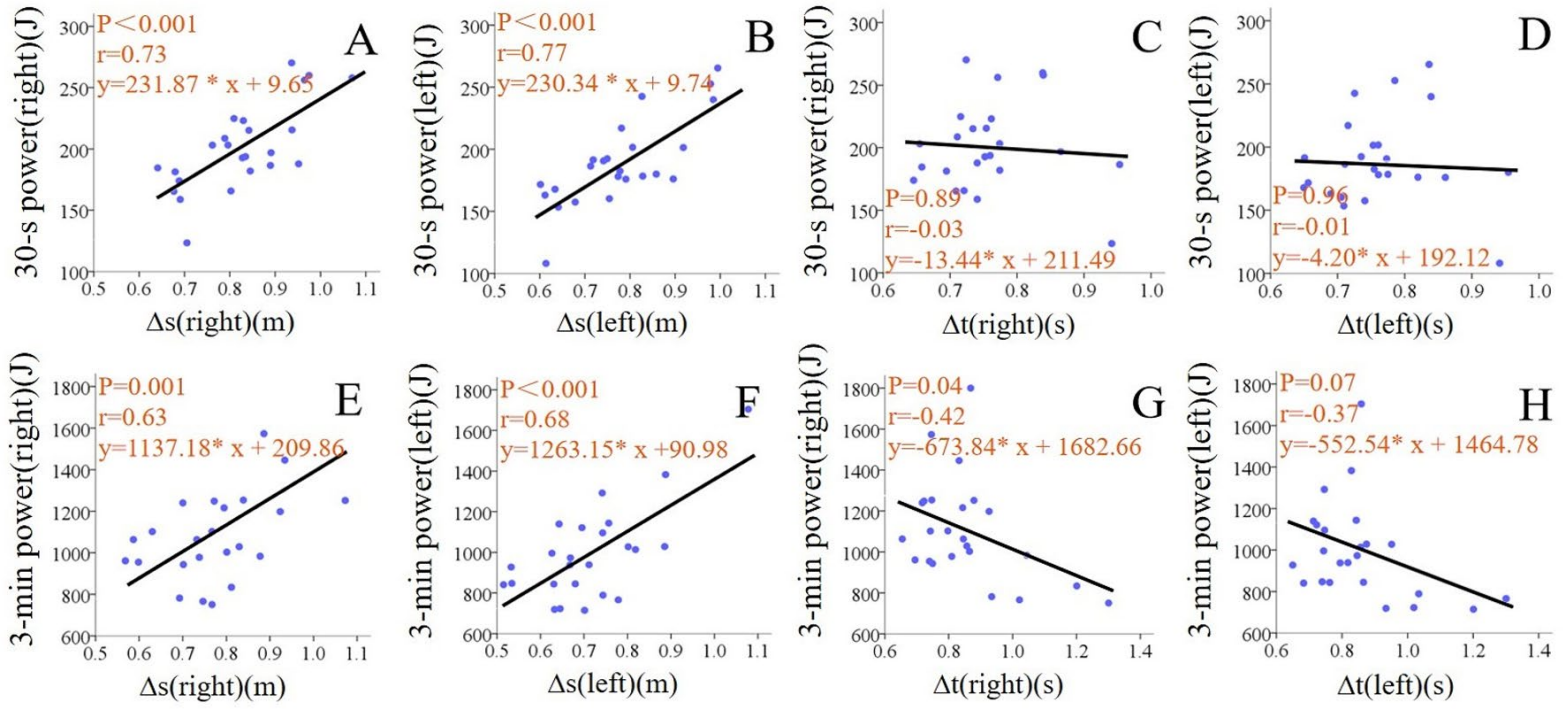
Correlations between ∆s, ∆t, and output power. Data from the left hand and right hand are calculated separately. **(A,B)** The relation between ∆s and 30-s output power. **(C,D)** The relation between ∆t and 30-s output power. **(E,F)** The relation between ∆s and 3-min output power. **(G,H)** The relation between ∆t and 3-min output power.

Pearson’s correlation coefficients between upper limb isokinetic muscle strength and subject performance (subject ∆s and output power in 30-s and 3-min) are shown in Table 2. Sit-skiing DP output power of 30-s was significantly correlated with isokinetic muscle extension strength of shoulder at all three different angular velocities of 30°/s (r = 0.39, *p* = 0.102), 60°/s (*r* = 0.61, *p* = 0.005) and 120°/s (*r* = 0.58, *p* = 0.01). Sit-skiing DP output power of 3-min was also significantly correlated with ioskinetic muscle extension strength of shoulder at all three different angular velocities of 30°/s (*r* = 0.47, *p* = 0.045), 60°/s (*r* = 0.66, *p* = 0.002) and 120°/s (*r* = 0.63, *p* = 0.004). There was no significant relation between isokinetic muscle strength of elbow and sit-skiing output power. ∆s of 30-s and 3-min were not significantly correlated with shoulder or elbow muscle extension strength. As shown in Fig. 1A,B and E,F, **∆**s was significantly and positively correlated with output power both in 30-s (right hand, *r* = 0.73, *p* < 0.001; left hand, *r* = 0.77, *p* < 0.001) and 3-min (right hand *r* = 0.63, *p* = 0.001; left hand *r* = 0.68, *p* < 0.001) test. Overall, **∆**t was not significantly correlated with the output power (Fig. 1C,D and G,H).

**Table 2:** Correlations between upper limb isokinetic muscle strength and subject performance (subject **∆**s and output power in 30-s and 3-min).

Pearson correlation	30-s ∆s (m)	3-min ∆s (m)	30-s power (J)	3-min power (J)
**Shoulder**				
Extend at 30°/s	0.243	0.115	0.387	0.465*
Extend at 60°/s	0.232	0.103	0.614**	0.656**
Extend at 120°/s	0.273	0.359	0.575**	0.626**
**Elbow**				
Extend at 30°/s	−0.22	−0.302	0.07	0.292
Extend at 60°/s	−0.345	−0.398	−0.008	0.171
Extend at 120°/s	−0.096	−0.111	0.289	0.434

Statistical significance is represented by **, *P* < 0.01, *, *P* < 0.05.

***∆****s* distance of a poling phase during double poling.

### Relationships between output power and upper limb isokinetic muscle strength

Multiple stepwise regression analysis between upper limb isokinetic muscle extension strength and the sit-skiing DP output power showed that muscle extension strength of shoulder at 60°/s accounted for 34% of the variance in the 30-s test, while extension strength of shoulder at 60°/s accounted for about 40% of the variance in 3 min test. Upper limb muscle extension strength measures from other velocities did not enter the regression.

## Discussion

The present results revealed that the shoulder extension strength was the main factor determining performance in DP. In detail, the muscle extension strength of shoulder at 60°/s accounted for 34% of the variance in the 30-s test, while the muscle extension strength of shoulder at 60°/s accounted for about 40% of the variance in the 3-min test. These findings add to an emerging body of literature explaining how upper limb muscle strength significantly contributes to the output power in cross-country sit-ski.

The correlation analysis showed that output power was significantly and positively correlated with upper limb isokinetic muscle strength. Correlation analysis of muscle strength and output power showed that shoulder isokinetic muscle extension strength at almost all angular velocities and modes were positively correlated with 30-s and 3-min output power, which is consistent with previous reports suggesting that the largest muscles of the shoulder, i.e., trapezius, deltoids, pectoralis major, and serratus anterior, are responsible for the majority of the upper-extremity tasks^5^. The muscle strength of elbow extension was not significantly correlated with output power count. One explanation for this situation might be that the participants in this work were all primarily skier, who mainly swung the upper arm during the DP process, thus making the elbow extension strength to contribute less. Also, our results supported this argument, i.e., our ∆s in a 3-min test was about 0.8 m, which is much smaller than in the non-disabled elite skiers, whose ∆s = 1.8–2.2 m^4^). As the participants in this study were not experienced in sit skiing, and thus may not have well developed motor strategy for producing propulsive force. Furthermore, as shown in Fig. 1A,B and E,F, larger ∆s corresponded to larger power output, thus highlighting the importance of elbow extension at the later stage of the poling. This is also consistent with the previous study reporting that arm swing considerably contributes to the overall force generation and propulsion^18^, i.e., as the arm swings more backward, the pole force sustains more efficiently and a longer time.

The above-listed correlations confirmed the importance of maximal strength training in cross-country skiers training programs^5^. Moreover, the same trend of lasting-mode (3-min) and short-mode (30-s) output power with the upper limb muscle strength were consistent with the results of non-disabled cross-country skiing UBP tests in the aerobic energy system^9^ and shorter sprint-type system^10^. These results highlight the importance of upper limb muscle strength for cross-country sit-skiing performance.

Stepwise multiple regression analysis of upper limb muscle strength showed that shoulder extension strength at 60°/s accounted for 34% of the variance in 30-s output power, while shoulder extension strength at 60°/s accounted for about 40% of the variance in 3-min output power (Table 3). The shoulder extending velocity of 60°/s was consistent with our kinematic analysis, where the shoulder was extended at about 55°/s during poling phase (calculated from markers sticking on the upper limbs, data not shown). In addition to a general increase in lean upper-body mass and maximal upper-body strength^15^, our results revealed the contribution of upper limb isokinetic muscle strength to the DP technique in cross-country sit-skiing.

**Table 3:** Unstandardized coefficient, standard error, partial correlation, and adjusted R^2^ of stepwise multiple regression analysis on the upper limb extended muscle strength and output power of 30-s and 3-min.

Predictor variables	B	SE	P-value*	r	Adjusted R^2^
**30-s output power**					
**Step 1**					0.341
Constant	110.417	29.554	0.002		
Shoulder extend at 60°/s	3.053	0.951	0.005	0.614	
**3-min output power**					
**Step 1**					0.397
Constant	348.706	212.651	0.119		
Shoulder extend at 60°/s	24.527	6.844	0.002	0.656	

*B* unstandardized coefficient, *SE* standard error, *r* partial correlation.

*All regression models were statistically significant (*P* < 0.05).

The above results indicated that upper limb isokinetic muscle strength and muscle coordination were important factors for the power output generation in sit-skiing DP. In detail, at the initial stage of the poling phase, the isokinetic extension strength at 60°/s of the shoulder muscle group dominated the poling action, and at the later stage of the poling phase, the elbow swing action was essential for enhancing the performance. These results may guide designing strength-training programs of cross-country sit-skiing athletes.

This study has a few limitations. Influenced by COVID-19, the researchers did not recruit as many participants as expected, let alone the professional athletes with lower limbs impairments. As the recruited participants were not experienced in sit- skiing, they may not have well developed motor strategy for producing propulsive force which preventing the contribution of elbow extensor muscle group to propulsive force. The non-disabled participants recruited in this study never skied in a sitting posture with their legs fixed. The poling posture and muscle activation^19^ of both upper limb and trunk are likely to be unstable and thereby influence the poling mechanics and output power. The researchers also did not consider longer testing of DP on the ergometer, which could make the testing more similar to the real situation of a cross-country sit-skiing race.

## Methods

### Participants

Among a total of 26 non-disabled students recruited from Capital University of Physical Education and Sports, 19 female students (age 23 ± 0.8 years, BMI 20.4 ± 2.2) finished all the tests. Inclusion criteria were as follows: participants who majored in physical training. Participants who could not finish the physical tests and isokinetic muscle strength measurements in 2 weeks or those suffering from injuries during the testing period were excluded from the study.

This study was approved by the Ethics Committee of the Capital University of Physical Education and Sports (Beijing, Peoples’ Republic of China), and all experiments were performed in accordance with relevant guidelines and regulations. All participants provided written informed consent prior to the enrolment. Individuals identifiable in the included images gave their informed consent for publication.

### Physical tests and experiment setups

**Figure 2:**
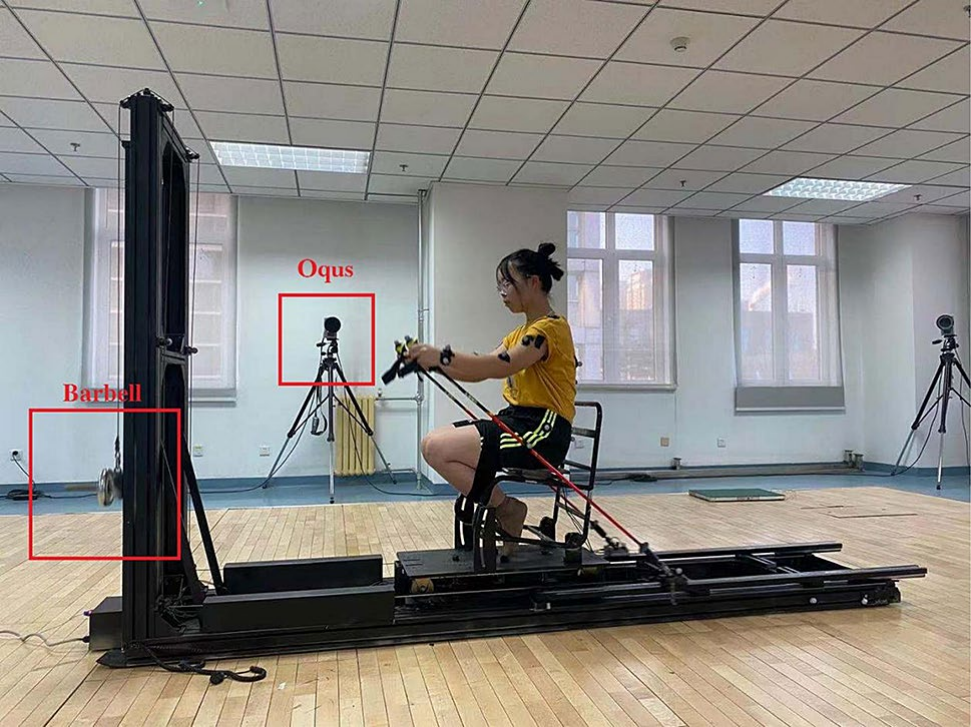
Illustration of the experimental setup during the simulation of cross-country sit-skiing double poling process.

All participants visited the lab twice to complete the doubling poling tests. Tests were performed on intelligent training and experimenting equipment for cross-country sit-skiing (Fig. 2)^20^ in the lab environment with a temperature of 23 ℃ and suitable humidity. During the first visit, participants were introduced to the testing equipment and allowed to warm up at self-selected resistance and poling rhythm. Following a 5 to 10 min warm-up, they were asked to sit with lower leg strapped to the seat so as to simulate the conditions of athletes with the disabled lower limb, after which they performed three successive 30-s maximal effort tests with 3 min rest in each group. Next, the participants were allowed to rest for 10 min before a final 3-min maximal effort test. Each test began with a 5-s countdown, during which participants were instructed to start poling with a slow but steady cadence. The poling resistance was set at 5% of body mass, which was chosen from several pilot testing and gave skiers the most natural feeling of double-poling. During the second visit, which took place no less than 24 h after the first visit, the participants were asked to repeat all the tests performed during the first visit.

The intelligent training and experimenting equipment of cross-country sit-skiing used the gravity force of barbell pieces as a substitute for normal skiing resistances. The device included an underframe and a vertical frame, where a training seat was installed on the underframe. Barbell pieces mounted on the light-weighted wheeled platforms moved on the guide rail of the vertical frame, thus forming a cable and pulley system. The cables were rerouted through the underframe and connected with the horizontal platforms. Cross-country ski poles, which were inserted into the top of the horizontal platforms, moved the platforms along the rails, thus enabling a simulated upper body double-poling motion. The horizontal rails could rotate around the proximal end by manually adjusting the fixing bolt. Elastic bands around the seat twining the thigh and ankle helped fix the legs, simulating the common situation in paralympic cross-country sit-skiing. As the skier pushed backward on both poles, the rope pulled the barbells sliding on the vertical rail against the gravity. Seven Oqus3 + cameras (Qualisys AB, Gothenburg, Sweden) were used to track the markers sticking on poling platform used to measure the barbell movement distance against the gravity direction. Marker motion data were recorded at 200 Hz.

${\text{Output powerduring thepoling cycle was defined as}}:{\text{ Output Power}}\, = \,\left( {{\text{barbell movement distance }}*{\text{ barbell gravity}}} \right)/{\text{time}}.$Following the typical definition of double poling^21^, a poling cycle consists of 2 main phases: the poling phase, during which the sled accelerates, and the recovery phase, during which the athlete gets ready for a new cycle. The total distance of the pole tip moves during the poling phase is named ∆s, and the corresponding time is named ∆t.

Isokinetic muscle strength of the shoulder and elbow was measured with the ISOMED2000 isokinetic system (Basic System and Back System; D&R Ferstl GmbH, Hanau, Germany). The tests were carried out in strict accordance with the standardized testing procedure. After 5 min warm-up on a rowing ergometer at a self-selected resistance, participant’s dominant limb was tested, and the shoulder and elbow were tested in a randomized order. Before the formal testing, participants were asked to perform a standard warm-up process consisting of five familiarization trials with submaximal concentric contraction efforts of the shoulder and elbow muscles at the angular speed of 60°/s. Verbal encouragement was provided with maximum effort during each test, and visual feedback from the computer screen was not permitted. The participant was fixed with straps, and the gravitational correction was applied after setting shoulder/elbow flexion and extension range of motion limits. The isokinetic concentric-concentric protocol was performed at 30°/s (3 repetitions), 60°/s (5 repetitions), and 180°/s (10 repetitions), and with a 120-s rest between different speeds^22^. Each testing condition consisted of three trials with a two-minute break in between. Maximum peak torque was taken, and only the maximum value was recorded.

### Statistical analysis

SPSS version 26.0 was used for all data analysis. Data normality was checked by the frequency distribution and probability-probability plots of the residuals. Values were recorded as mean values ± standard deviations (SD). Associations among technical performance, output power, and muscle strength were explored by Pearson’s correlations. Stepwise multiple regression on output power measurements was used to identify significant predictive variables from measures of muscle strength of different testing modes and muscle groups. P-values ≤ 0.05 (two-sided) were considered statistically significant in all analyses.
